# Effectiveness of longitudinal faculty development programs on MCQs items writing skills: A follow-up study

**DOI:** 10.1371/journal.pone.0185895

**Published:** 2017-10-10

**Authors:** Hamza Mohammad Abdulghani, Mohammad Irshad, Shafiul Haque, Tauseef Ahmad, Kamran Sattar, Mahmoud Salah Khalil

**Affiliations:** 1 Department of Medical Education, College of Medicine, King Saud University, Riyadh, Saudi Arabia; 2 Oral Microbiology Laboratory, Department of Bioclinical Sciences, Faculty of Dentistry, Health Sciences Centre, Kuwait University, Kuwait; 3 Research and Scientific Studies Unit, College of Nursing and Allied Health Sciences, Jazan University, Jazan, Saudi Arabia; Kyoto University, JAPAN

## Abstract

This study examines the long-term impact of the faculty development programs on the multiple choice question (MCQ) items’ quality leading to study its effect on the students’ overall competency level during their yearly academic assessment. A series of longitudinal highly constructed faculty development workshops were conducted to improve the quality of the MCQs items writing skills. A total of 2207 MCQs were constructed by 58 participants for the assessment of 882 students’ cognitive competency level during the academic years 2012–2015. The MCQs were analyzed for the difficulty index (P-value), discriminating index (DI), presence/absence of item writing flaws (IWFs), and non-functioning distractors (NFDs), Bloom’s taxonomy cognitive levels, test reliability, and the rate of students’ scoring. Significant improvement in the difficulty index and DI were noticed during each successive academic year. Easy and poor discriminating questions, NFDs and IWFs were decreased significantly, whereas distractor efficiency (DE) mean score and high cognitive level (K2) questions were increased substantially during the each successive academic year. Improved MCQs’ quality leaded to increased competency level of the borderline students. Overall, the longitudinal faculty development workshops help in improving the quality of the MCQs items writing skills of the faculty that leads to students’ high competency levels.

## Introduction

Effective delivery of medical science education demands precise and efficient assessment system(s) capable of examining students’ essential subjective knowledge, learning attitude and practical skills at high competency level. The assessment of high level competency changes students’ learning behavior and ingrains enthusiasm in the students to accumulate subject’s explicit and implicit information from the lecturers or other available resources [1–3]. In the assessment system, the use of multiple choice questions (MCQs) is a very common and well accepted method of evaluating diverse characteristics of the professional competencies of medical science education [4]. Moreover, medical education agencies are adopting the use of MCQs in the assessment system due to several advantages over the other assessment formats [5,6]. Most importantly, MCQs based assessment system is being used to test the cognitive competencies of a large number of students at-a-time with a broad range of curriculum content along with its objectivity, higher reliability, validity and ease of scoring [7,8]. Earlier studies have reported that MCQs are superior and apposite competency test for evaluating the subject’s knowledge, comprehension, and can be designed to examine applications and analyses [9]. Construction of high quality MCQs can test advanced level critical thinking and application of the knowledge by evaluating the examinee’s ability to integrate, synthesize and evaluate medical information [10–12]. Use of high quality MCQs in the assessment process determines the deep learning approach(s) of the students towards higher Bloom’s taxonomical level of cognitive abilities such as interpretation, synthesis and application of the acquired knowledge instead of testing the recalling of the isolated facts [13].

Construction of high quality MCQ items is a very challenging task for the faculty members, especially for those who have never undergone precise and dedicated training [14,15]. However, MCQ items writing practice guidance has been well documented in the literature since long to guide the faculties who wish to construct MCQ items meeting with the standard assessment format [16–18]. Regardless of the availability of MCQ items writing guidelines, many reports have been published stating about various deficiencies and flaws in MCQ items of medical tests [17–20]. Deviations from MCQ items writing guidelines generally result in undesirable changes of the items’ statistical factors like discrimination index (DI), difficulty index (P-value), validity of the examination, and percentage of students’ score [19,21,22]. The potential factors, such as lack of familiarity with MCQ items writing guidelines, reluctance towards change in personal writing habits, and lack of comparative experimental data based on the performance in the examination, generally contribute for low acceptance of MCQ items writing guidelines and its applications among the faculty members [23].

Only scanty studies have reported the introduction of faculty development programs dedicated to train the faculties for the construction of MCQ items or to improve the items’ writing skills to develop high quality MCQ items [14,15,24]. Several studies mentioned faculty development programs dealing with self-reported participants’ perception of learning and behavioral change during quality evaluation of the test items [25,26]. A large number of studies suggested the need of dedicated longitudinal academic development programs delivered by medical education experts for better participation of the participants in terms of practice, reflection, feedback and improvement [27–30]. Also, proficient evaluation of the academic programs is one of the essential educational process of determining whether the program objectives have been achieved by the participants at their efficiency level or not [31].

**Fig 1:**
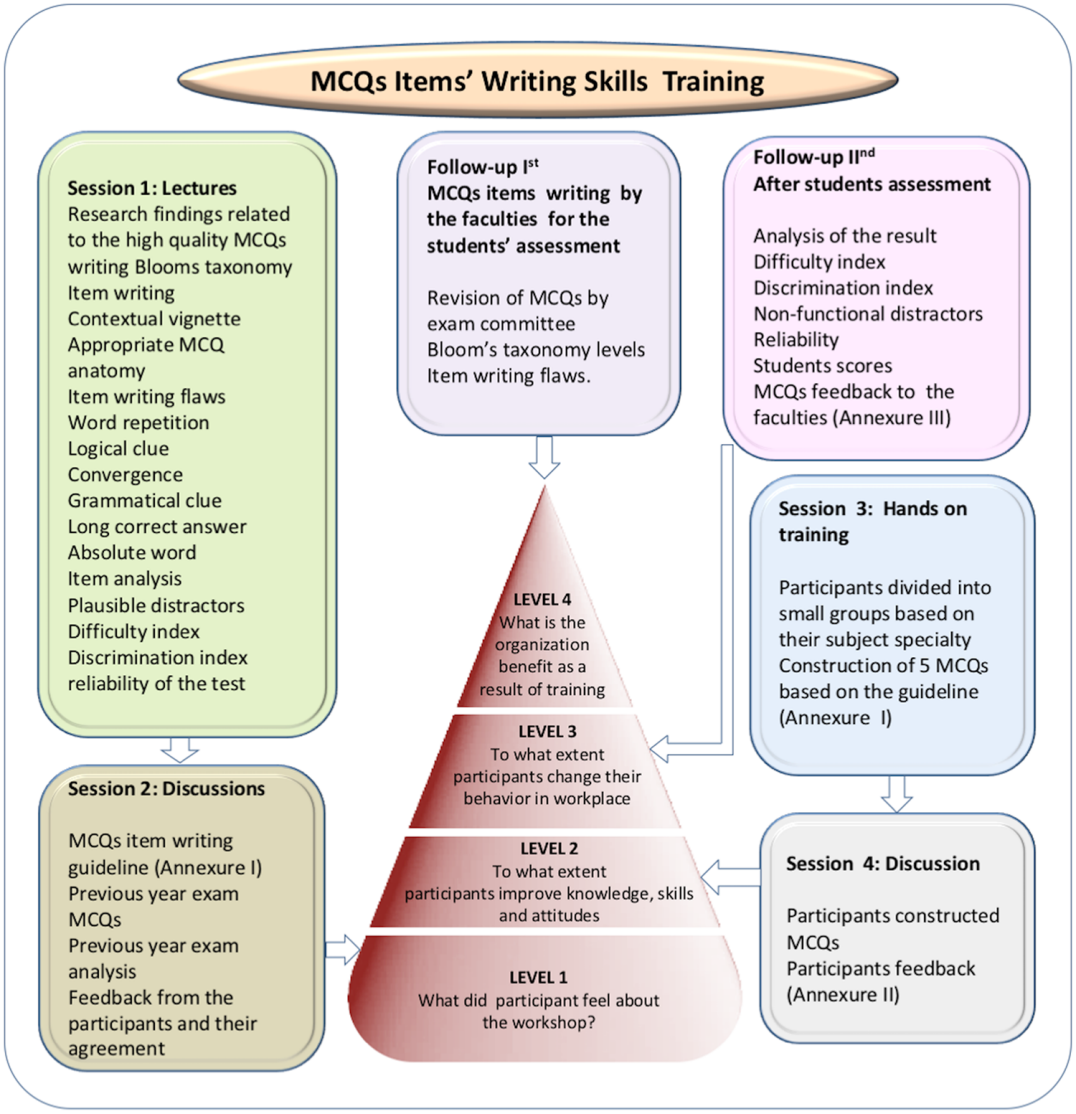
MCQs items writing training workshop program structure (adopted from Abdulghani et al., 2015).

The Assessment and Evaluation Center (AEC) of medical education department, college of medicine (COM), King Saud University (KSU), Riyadh, Saudi Arabia has introduced longitudinal faculty development program for MCQ items writing workshop training for one day during each academic session in order to assist the faculty members in their academic roles. The AEC has developed a well-designed faculty development workshops training model dedicated for MCQ items writing. A schematic representation of the longitudinal faculty development program’s model introduced at the college of medicine, KSU, Riyadh, Saudi Arabia, has been given in Fig 1. The suggested model efficiently evaluates the participants’ reaction, level of acquired knowledge and skills, changes in the practice, application of the learning to practice, and changes in the levels of the learner and the organization, as the main outcome of the program was perception, which corresponded to the fourth level of Kirkpatrick’s model [14]. This is the extension and follow-up study of our previously published research work, where we retrospectively examined the effects of long term systematic pre- and post-faculty development workshop training programs to the newly joined faculty members, in order to improve their quality of MCQ items’ writing skills [14]. In the previous study, MCQ items were analyzed for difficulty index (DI), discriminating index, reliability, Bloom’s cognitive levels, item writing flaws (IWFs) and MCQs’ nonfunctioning distractors (NFDs), and significant improvements were found between pre- to post-training [14]. In the present study, we have examined the effects of longitudinal faculty development workshop training on MCQ test items writing outcomes and its influence on overall performance of the students.

## Methods

### Study context

The College of Medicine (COM), King Saud University (KSU), Riyadh, Saudi Arabia is the first and the most premier medical college of Saudi Arabia. Based upon its international ranking (http://www.shanghairanking.com/World-University-Rankings/King-Saud-University.html; last accessed on 28.12.2016), it has to meet the global medical education standards and accreditations [32]. Therefore, the department of medical education was created to review and maintain the quality standards of medical science education at different levels, including curriculum reform, organizing pedagogical trainings and applying novel and innovative pedagogical strategies, and implementing apposite assessment systems in order to achieve the highest standard level. The COM, KSU offers a Bachelor's of Medicine and Surgery (MBBS) degree program which follows a system based integrated curriculum distributed over the five years. The first two years are pre-clinical years dealing with basic science subjects of clinical relevance in the form of block systems of gastrointestinal, respiratory, reproductive, cardiovascular, musculoskeletal, renal, endocrine and nervous system. Third year is the introductory year for the clinical subjects and includes medicine, surgery, epidemiology, and research skills. Fourth year (clinical) comprises subjects of anesthesia, ENT, dermatology, ophthalmology, orthopedics, primary health care, Obstetrics/Gynecology and psychiatry. The fifth year (clinical) deals with medicine part-2, pediatrics, and surgery part-2 in addition to four-weeks of elective course including rotations and training in the hospital in all the required disciplines to complete the clinical internship requirements.

### Participants

A total of 58 faculty members were involved in the teaching and assessment of the first and second year courses during the academic years 2012–2015. During each academic year, the faculty members were requested to submit the MCQs for the students’ evaluation. The faculty members were instructed accordingly to follow the MCQs’ construction checklist (Supporting Information: S1 Appendix) for the items writing [14]. A standard guideline for the construction of high quality MCQs was already developed by the Assessment and Examination Center (AEC), COM, KSU, and the same was implemented for the construction of MCQs (Supporting Information: S1 Appendix) [14]. The MCQs of the final exam were reviewed by the examination committee members of the AEC to assure the quality standard (Supporting Information: S2 Appendix).

### Intervention of a longitudinal MCQs items writing workshop

In order to write a high quality MCQs items and to fulfill the uniform standards of the MCQs construction checklist, the AEC, COM, KSU, organizes high standard longitudinal faculty development FD workshop training since the academic year 2012. In general, the AEC organizes one workshop in each semester, i.e., two workshops per academic year. The workshops were conducted for one-day full-time and the faculty members of the COM, and other allied healthcare colleges of KSU were invited to participate in the MCQs items writing training. The main aim of the workshops was to improve the MCQs construction skills of the faculty members. The first two hours of the workshop dealt with theoretical background, importance of high quality MCQ items development along with the revision of the MCQs construction checklist criteria and critical discussion of the previous examination MCQs. A consensus was achieved regarding the MCQs construction checklist items with the participating AEC members. In case of a disagreement on any item mentioned in the checklist, it was openly debated for its rationale and discord was resolved. Additionally, in case of failure or inconclusive situation of the above stated open debate, the matter was resolved with the help of an adjudicator (Supporting Information: S1 Appendix).

During the remaining time of the workshop, the participants were divided into small groups of three to four participants and task of five MCQs construction in their specialties was given following the agreed checklist criteria. Further, the constructed MCQs were discussed, corrected and edited with the participants’ agreement. A schematic representation of the well-structured MCQs items writing workshop program as a flow-diagram has been given in Fig 1.

### Follow–up studies of the longitudinal workshop

The MCQ test items were constructed by the faculty members for the first and second year (pre-clinical year) students’ final examinations. The test items were prepared for all the eight courses of gastrointestinal, respiratory, reproductive, cardiovascular, musculoskeletal, renal, endocrine system and nervous system following the MCQs construction checklist criteria (Supporting Information: S1 Appendix). Before the students’ assessment, MCQs were critically discussed in the examination board comprising of various faculty members. After the examination, the scoring-rate of each question was discussed in faculty development workshop training program and error(s) occur during the MCQ item construction was further corrected in the next academic year.

In this study, the MCQ items written by the academic faculties during the academic years 2012–2013, 2013–2014 and 2014–2015 were considered and included for the quality assessment. A total of 2207 MCQ items were used to measure the main outcomes, The quality of the MCQ items was evaluated in terms of construction (Bloom's taxonomic cognitive levels and presence/absence of the item writing flaws (IWFs)), MCQs items analysis ((Difficulty index (P), Discriminating index (DI), non-functioning distractors (NFDs), and test reliability (Kr-20)), and student's performance (mean score and overall passing rate). Question Mark Perception Software Program (Question Mark Corporation, Norwalk, CT, USA) was used for the items’ analysis and for the determination of the test reliability. The present study lies in the fourth level of the Kirkpatrick’s model, which evaluates the changes among the participants’ performance based on the MCQ items writing outcomes at three different levels.

### MCQs items construction in terms of Bloom's cognitive level and item writing flaws

A well-constructed MCQ consists of a stem (a clinical case scenario), a lead-in (question), followed by four choice options (one correct/best answer and remaining three, distractors) [33,34]. Bloom's taxonomy divides the cognitive domains into six hierarchically ordered categories, i.e., knowledge, comprehension, application, analysis, synthesis, and evaluation [35]. Tarrant et al., [36] simplified the taxonomy by creating two different levels, i.e., K1, which represents the basic knowledge and comprehension, whereas K2 encompasses application and analysis. Generally, the MCQs with IWFs are those items that violate the standards suggested by the item-writing guidelines [17]. In order to measure the effectiveness of the faculty development program, a checklist was prepared (based upon the consensus of the faculty members during the multiple faculty development workshop trainings) for evaluating the quality of the MCQs (Supporting Information: S1 Appendix).

### MCQs items analysis in terms of difficulty index, discrimination index, non-functional distractors, and Kr-20

Difficulty index also termed as P-value, describes the percentage of students who answered correctly in response to a given test item. The difficulty index ranges between 0 to 100% or 0 to 1. An easy item has a higher difficulty index value. The cut-off values for the evaluation of the difficulty index of the MCQs were >70% (easy); 20–70% (moderate); and <20% (difficult).

Discriminating index (DI) is the ability of a test item to discriminate between high and low scoring examinees. Higher discriminating indices of a test indicate better and greater distinguishing competence of the test. The cut-off values for the DI were taken as: DI > 0.15, which considered as highly discriminating, and low or Non-DI as ≤ 0.15 [37].

Non-functioning distractor(s) (NFD) is/are an option(s) of a question other than correct answer and generally reported that less than 5% of the examinees used to select the NFDs [22]. The NFDs may have no connection or have some relevance that are not directly related to the correct answer [36]. Implausible distractors can be easily spotted even by the weak examinees and are therefore usually rejected straightaway. Distractors that are not chosen or are consistently chosen by only few participants are obviously ineffective and must be replaced or omitted [38,39].

Distractor efficiency (DE) is determined for each MCQ item on the basis of the number of presence or absence of NFDs, and ranges from zero to 100%. If a MCQ item contains 0-NFD, 1-NFD, 2-NFD or 3-NFD then it corresponds to DE of 100, 66.6, 33.3 or 0%, respectively [22]. The Kuder-Richardson Formula 20 (Kr-20) measures the internal consistency and reliability of an examination. The Kr-20 formula is a measure of internal consistency of the examinations with dichotomous choices. High Kr-20 coefficient (e.g., >0.90) indicates homogeneous test items. The Kr-20 value 0.8 represents the optimal acceptable test items, while the Kr-20 values below 0.8 suggests poor reliability of the test [40].

### Students’ assessment and their performance

The MCQ items writing flaws and plausible distractors might severely affect students’ performance. Some items writing flaws such as the use of unfocused or unclear stems, gratuitous or unnecessary information and use of negative wordings in the stem can make the MCQ even more difficult [19]. Likewise, plausible distractor creates misconception about the correct option especially for the borderline students [41].

### Statistical analysis

The data obtained were entered in the Microsoft Office Excel file and analyzed using SPSS software (version 22.0). Pearson's chi-square test was used to evaluate and quantify the correlation. The statistical significance level was maintained as p-value < 0.05 during the entire statistical analysis.

### Ethical considerations

The participants were informed about the study and were agreed to get involved in the project. The study was approved by the research ethical committee of the COM, KSU, Riyadh, Saudi Arabia. In addition, the methods employed in this study were carried out in accordance with the approved guidelines of the college.

## Results

A total of 2207 MCQ items were prepared by the faculty members of COM, KSU. Out of 2207 MCQ items, 729 MCQs were constructed during the academic year 2012–2013, 690 MCQs were constructed in the academic year 2013–2014, and 788 MCQs were constructed in the academic year 2014–2015. These MCQ items were used for the assessment of the studied courses of gastrointestinal, respiratory, reproductive, cardiovascular, musculoskeletal, renal, endocrine system and nervous system. The MCQ items were prepared according to the guidelines of AEC, COM, KSU (Supporting Information: S1 Appendix). Our results examined the reliability co-efficient (i.e., Kr-20) of MCQ items of all the eight courses and they were found to be ≥ 0.88 in the academic year 2012–2013, ≥ 0.90 in the academic year 2013–2014, and ≥ 0.92 in the academic year 2014–2015 (Table 1). While, students’ mean score was decreased as 80.18, 78.06 and 74.32% during the academic years 2012–2013, 2013–2014 and 2014–2015, respectively.

**Table 1:** Description of the examinations.

Parameters	Academic years		
	2012–2013	2013–2014	2014–2015
Total MCQs	729	690	788
Total distractors	1370	1263	1272
FD, n(%)	109(7.95)	126(9.97)	234(18.39)
NFD, n(%)	1261(92.05)	1137(90.03)	1038(81.61)
P-value ((mean(SD)	80.35(14.24)	78.19(17.08)	74.16(18.2)
DI ((mean(SD))	0.38(0.12)	0.37(0.12)	0.37(0.11)
DE ((mean(SD)	42.31(34.92)	45.04(35.76)	56.06(36.20)
Test Reliability (Kr-20)	0.88–0.94	0.90–0.94	0.92–0.94
Scoring rate((mean(SD)	80.18(4.12)	78.06(5.37)	74.32(5.43)
Passing rate (%)	88.37	85.34	78.74

FD, functional distractors; NFD, non-functional distractors; DI, discriminating factors; DE, distractor efficiency; SD, standard deviation

A comparative analysis was performed for the MCQ items of all the eight courses studied during the above mentioned three academic years. The percentage of easy questions’ difficulty index (P-value >70%) was decreased and the percentage of moderate questions (P-value = 20–70%) was increased during each successive academic year. The results depicted that the P-value was significantly improved during each successive academic year (χ^2^ = 30.02; p = 0.0001). Likewise, the quality (i.e., good construction) of the constructed MCQ items was assessed with the help of discrimination index (DI) values. The MCQ items’ DI values were found proportional of good/poor constructed questions ratio (%) and it was 87.8/12.2 during the academic year 2012–2013. Interestingly, the said DI values were increased to 90.0/10.0 during the academic year 2013–2014, and 91.6/8.4 during the academic year 2014–2015 (χ^2^ = 6.12; p = 0.047). Further analysis of the MCQ items revealed a significant increase in the questions with functional distractors. However, a significant decrease was noticed in the number of questions having non-functional distractors (3-NFD) during each successive academic year (χ^2^ = 67.92; p = 0.0001). The proportional of FDs and 3-NFDs during the academic year 2012–2013 were 15.0 and 30.0%, during the academic year 2013–2014 were 18.3 and 27.8%, and during the academic year 2014–2015 were 29.7 and 18.4%, respectively.

On the basis of Bloom’s cognitive levels, during the academic year 2012–2013 the K1 level MCQs were more (73.3%) in the number as compared to the K2 level MCQs (26.7%). But, during the academic year 2013–2014, the K1 level MCQs were decreased (73.2%) in the number as compared with the K2 level MCQs (26.8%). Whereas, during the academic year 2014–2015, the K2 level MCQs were increased (31.2%) in comparison with the previous year questions (K1 MCQs, 68.8%) (χ^2^ = 4.91; p = 0.086). A statistically significant decrease in the item writing flaws (IWFs) was also witnessed during each successive year (χ^2^ = 20.87; p = 0.001) (Table 2).

**Table 2:** Factors associated with the MCQs items analysis.

Factors	Categories	Academic years			χ^2^, p
		2012–2013 n(%)	2013–2014 n(%)	2014–2015 n(%)	
Difficulty Index (P)	Difficult (<20)	2(0.3)	7(1.0)	10(1.3)	30.02
	Moderate(20–70)	142(19.5)	166(24.1)	239(30.3)	0.0001
	Easy (>70)	585(80.2)	517(74.9)	539(68.4)	
Discriminating Index (DI)	DI>0.15	640(87.8)	621(90.0)	722(91.6)	6.12
	D1≤0.15	89(12.2)	69(10.0)	66(8.4)	0.047
Non-functional distractors (NFDs)	FD	109(15.0)	126(18.3)	234(29.7)	67.92
	1NFD	198(27.2)	183(26.5)	215(27.3)	0.0001
	2NFD	203(27.8)	189(27.4)	194(24.6)	
	3NFD	219(30.0)	192(27.8)	145(18.4)	
Item writing flaws (IWFs)	Non-IWFs	667(91.5)	649(94.1)	764(97.0)	20.87
	IWFs	62(8.5)	41(5.9)	24(3.0)	0.001
Blooms’ taxonomy levels	K1	534(73.3)	505(73.2)	542(68.8)	4.91
	K2	195(26.7)	185(26.8)	246(31.2)	0.086

The results of cross analysis revealed that the presence of a high percentage of NFDs affect the DI and P-values. The presence of a high percentage of 3-NFDs in the MCQs enhanced the ease of a question (χ^2^ = 816.5; p = 0.0001), also the discriminating power between the high/low achieving students (χ^2^ = 14.34; p = 0.002) (Table 3). No statistically significant correlation was observed between the NFD and IWFs (χ^2^ = 2.26; p = 0.519). Interestingly, the MCQs with IWFs scored higher than the MCQs without IWFs. Likewise, difficult MCQs scored lower than the moderate or easy questions (Table 4). Also, the students’ scoring rate was significantly influenced by the proportional of FD or NFD questions as observed over all the three academic years. The students’ mean score (± SD) of the FD questions was 59.17 ± 15.14, while the students’ mean score of 1-NFD questions was 71.76 ± 13.70; the mean score of 2-NFD questions was 82.82 ± 9.56, and the mean score of 3-NFD question was 93.37 ± 5.77. The overall results revealed that a high percentage of NFD questions may contribute to the students’ high scoring rate that lead to a significant increase in the number of borderline students scoring high.

**Table 3:** Effect of distractors on difficulty and discrimination indices and item writing flaws.

Distractors (DE%)	Difficulty Index (P)				Discriminating Index (DI)			Items writing flaws		
	Difficult n(%)	Moderate n(%)	Easy n(%)	χ^2^, p	DI n(%)	Non-D1 n(%)	χ^2^, p	Without-IWFs n(%)	IWFs n(%)	χ^2^, p
FD (100)	11(2.3)	324(69.1)	143(28.6)	816.5	431(91.9)	38(8.1)	14.34	443(94.5)	26(5.5)	2.26
1NFD (66.6%)	6(1.0)	181(30.4)	409(68.6)	0.0001	546(91.6)	50(8.4)	0.002	564(94.6)	32(5.4)	0.519
2NFD (33.3%)	2(0.3)	39(6.7)	545(93.0)		529(90.3)	57(9.7)		556(94.9)	30(5.1)	
3NFD (0%)	0(0.0)	3(0.5)	553(99.5)		477(85.8)	79(14.2)		517(93.0)	39(7.0)	
Total	19(0.9)	547(24.8)	1641(74.4)		1983(89.9)	224(10.1)		2080(94.2)	127(5.8)	

**Table 4:** Different factors associated with the students scoring rate.

Factors	Categories	Mean score (SD)	ANOVA (p)
Distractors	FD	59.17(15.14)	0.0001
	1NFD	71.76(13.7)	
	2NFD	82.82(9.56)	
	3NFD	93.37(5.77)	
IWFs	No	78.30(15.16)	0.0001
	Yes	63.84(27.11)	
Difficulty level	Difficult	14.58(4.67)	0.0001
	Moderate	55.40(11.70)	
	Easy	85.55(7.83)	

## Discussion

The violation of the MCQ construction guidelines have been observed by the AEC examination committee members (unpublished data). The present study followed the continuous faculty development program held at COM, KSU twice per year, with rigorous review, follow-up and feedback from the faculty members that significantly improved their ability for high quality MCQ construction. In this study we tried to report the importance of continuous longitudinal faculty development programs for MCQ items skills development conducted in each semester of the academic session and their impact on MCQs’ quality leading to effect on students’ competency level.

Faculty development training workshops have significant impact on the participant’s MCQs item writing skills as demonstrated by improved test item’s quality revealed by the students’ assessment during each academic year, and the items’ quality was continuously progressing in the successive academic years. The reactions of the participants of the faculty development training workshop program reflected that they became more experienced and skilled in constructing high quality MCQ items after attending the workshop. The final results obtained from the students’ assessment, and the scoring rate of each item suggests that the longitudinal training helps the faculty members to renew or upgrade their academic skills [28,42]. Improved quality of the students’ assessment inferred that the participants have respect, desire, and support for the learning, and thus differentiate the under boarder line students achievement in each academic year. The results suggest that the longitudinal faculty development programs are effective and have long lasting and even some times indelible impact on the retention of the knowledge and skills in their current academic setting [43,44]. Likewise, the longitudinal MCQ items writing programs lead to continuous improvement of the MCQs test item’s quality according to the items writing checklist during each academic year. Earlier studies explored the participants’ skills feedback, immediately after the faculty training program [15,26]. On the similar lines, in the present study we found participants’ positive feedback immediately after the MCQs items writing workshop. Also, most importantly we did follow-up study to examine the long-term impact of the longitudinal faculty development program of MCQs item writing. Earlier, we have reported the introduction of systematic, organized, and dedicated faculty development program to train the faculties for the construction of high quality MCQ items or to improve their items’ writing skills for the construction of high quality MCQs items [14]. In continuation with our earlier study, this is the follow-up report of our previous findings/suggestions to evaluate the impact of the formerly introduced MCQ items writing training workshop. This is the very first study reporting the substantial long term impacts of the longitudinal faculty development program of MCQ items writing skills development. Although, numerous studies have reported the long term outcomes of the longitudinal academic program in the teaching and curriculum skills development in the medical sciences, but none of them is dedicated for studying the impacts of the MCQ items writing skills training workshops [45,46].

Various studies have reviewed the MCQs test items used for the students’ assessment in the past and found a high percentage of items writing flaws (IWFs) and non-functional distractors (NFD) [18,22,47]. Due to the presence of above mentioned and other destructive factors in the MCQs test items, dedicated MCQ test items writing training by the medical expert was recommended by the above authors. Our study is in agreement with the above authors’ recommendation, because several important productive feedbacks were observed in the MCQs’ test items that might be attributed to the well-constructed longitudinal faculty development program. The overall results revealed that the FD training helps in the improvement of the quality of the MCQs in terms of difficulty index (P-value) and discriminating indices (DI), Bloom’s cognitive levels and reduced item writing flaws (IWFs), and non-functioning distractors (NFDs). The presence of a high percentage of flaws items in a test reduces the validity of the exam(s) and penalizes the examinees [17]. Similarly, the plausible distractor creates misconception about the correct option, at least in the average examinee's mind [14]. Although, the MCQ items at K2 level are always better, more valid and capable of discriminating the good students from the poor once [48]. A high percentage of modestly difficult items in a test have better discriminating ability [49,50]. The IWFs generally violate the standard item-writing guidelines that affect the students’ performance and make the MCQ items either very easy or sometimes even more difficult [14]. Thus, IWFs present in the MCQs generally interfere with the accurate and meaningful interpretation of the students’ test scores and have a negative effect on the high-achieving students and their passing rates [19,51]. A high percentage of NFD increases the borderline students’ scoring and their passing rate [14]. Many authors have reported that the MCQ items with a higher number of NFDs are easier than those with a lower number of NFDs and are less discriminatory in nature [14,20,37]. Distractors usually failed to mislead the knowledgeable examinees as they don’t form any tricky question, but they got full success in distracting the less knowledgeable students [14]. A question with only two good distractors, however, is preferable to one with additional filler option added only to make up some pre-determined number of the options [52]. An effective distractor will look plausible to the less knowledgeable students and lure them away from the correct option, but failed to entice the students who are well informed about the topic under consideration [14].

The results of the present follow-up study were consistent with our previous report, in which the same model of the faculty development workshop training program was performed for the newly joined faculty members of the College of Medicine, Princess Nourah University, Riyadh, Saudi Arabia, and found that the quality of the MCQ items was improved significantly when compared with the pre- and post-training MCQs test items [14]. Overall, the writing of correct and effective MCQ items’ stem (clinical scenario), lead-in (question) and distractors is a challenging job, but helpful guidelines or systematic and repeated training can make the process easier and improved the MCQs Items’ quality. Ideal MCQs items adequately assess the students’ performances during the exams and affects the students’ grade, help in providing better career opportunities and aid in securing future educational scholarship or research fellowships. On the other hand, inaccurate assessment of the students’ competency drastically affects the overall career path of the students, and also pose a serious question about the reliability and legitimacy of the academic organization in the broader terms.

This follow-up study is unique in a sense, especially because the longitudinal faculty development program was primarily focused on the assessment of the quality. Overall, this follow-up survey evaluated the faculty development training program’s success rate in terms of MCQ test items’ quality, validity of the examination, and the assessment of students. The comparative analysis of the results of the students’ assessment revealed that the longitudinal model workshop/training for the faculty development is an effective and excellent strategy in the educational setting.

## Conclusion

Well-constructed longitudinal faculty development workshop trainings aid to improve the quality of MCQs items writing skills in terms of discriminating and difficulty indices as evident from the Bloom’s taxonomy cognitive levels, reduced item writing flaws, and increased functioning distractors. Improvement in the quality of the MCQs might endorse with the validity of the examination, better achievement of the students, and discrimination in the competency level of the borderline students. Based upon the outcomes, the present follow-up study suggests that the longitudinal faculty development programs need active participation of the faculty members, as these programs help in the improvement of the quality of the medical science MCQs’ writing that ultimately leads to higher competency level of the students.

## Supporting information

S1 Appendix Guidelines for avoiding the common item writing flaws in multiple-choice questions (adopted from Abdulghani et al., 2015). (DOCX)

S2 Appendix Examination feedback. (DOCX)

S1 Datapoints Individual datapoints gathered/used during the study. (XLSX)
