# Supplementary material for: Effectiveness of longitudinal faculty development programs on MCQs items writing skills: A follow-up study
Source: PLoS One. 2017 Oct 10;12(10):e0185895. doi: 10.1371/journal.pone.0185895 (PMC5634605; doi:10.1371/journal.pone.0185895)
Supplement: S1 Appendix — (DOCX) [file pone.0185895.s001.docx]

**Supporting Information: S1 Appendix**

**Guidelines for avoiding the common item writing flaws in multiple-choice questions (adopted from Abdulghani et al., 2015)**

1. In pre-clinical 40% of total MCQs and in clinical phases 70-80% or all of total MCQs should be Scenario based and the scenario should be related to the question.
2. Avoid negative questions. e.g., Which ONE of the following is NOT a characteristic...
3. No "EXCEPT" marked question.
4. Avoid usage of ambiguous (e.g., frequently, often, occasionally) or absolute terms (e.g. almost, never, frequent).
5. Options do not give clue to the answer (a word in the stem repeated in the option(s)).
6. All options are uniform/homogenous e.g. Identification/Diagnosis/Management as a separate entity in each question.
7. All options must be short and of a similar length. A single long option should not be a correct answer, which gives the clue of being the true answer.
8. Avoid ‘all of the above' or ‘none of the above' option
9. Put the options in a chronological order; i.e. ascending/descending if numerical, and alphabetical otherwise.
10. Use questions and avoid phrases. e.g. Regarding epilepsy:
11. Should have only one correct answer.
12. Should fulfill the cover test, i.e. if you cover the options, you can answer the questions (the single most important criteria for a good MCQ).
13. Options shouldn’t be overlapped.
14. Stem should be clear.
15. Lead in should be clear.
16. Answer should not hinge to another question.
17. Avoid True or False.
18. Scenario should be functional.
19. Format of question is shown below:

| **SCENARIO** | |
| --- | --- |
| **Lead in (Question)** | |
| 2. (**Options)** |  |
