# Supplementary material for: Effectiveness of longitudinal faculty development programs on MCQs items writing skills: A follow-up study
Source: PLoS One. 2017 Oct 10;12(10):e0185895. doi: 10.1371/journal.pone.0185895 (PMC5634605; doi:10.1371/journal.pone.0185895)
Supplement: S2 Appendix — (DOCX) [file pone.0185895.s002.docx]

**Dear colleagues**

Please find the post-exam feedback form based on the MCQs analysis of the course (course code). Please review the MCQs analysis for further improvement of MCQs item writing and student assessment system.

| Course |  | Students appeared |  |
| --- | --- | --- | --- |
| Exam |  | Students passed |  |
| Date |  | Students failed |  |
| No. of questions |  | Maximum marks |  |
| Total marks |  | Minimum marks |  |
| Test Reliability Index (KR20) |  | Mean score (%) |  |

| **Difficulty level** | **No. of question** | **Criteria** |
| --- | --- | --- |
| Easy question |  | ≥70% – 89% students answered correctly |
| Moderate questions |  | 21% - 69% students answered correctly |
| Hard question |  | ≤ 20% students answered correctly |

| **Difficulty level** | **Q. No.** | **Corrected by students (%)** |
| --- | --- | --- |
| Easy questions |  |  |
| Difficult questions |  |  |
|  |  |  |

| **Q. No.** | **Point biserial** | **Corrected by students (%)** | **Inference** |
| --- | --- | --- | --- |
|  |  |  | Wrong key |
|  |  |  | Easy question |
|  |  |  | Implausible options |

**Reliability level:**

Excellent/ Very good/ Good/ Some what low/ Low reliability.

**Overall students’ performance:**

Excellent/ Very good/ Good/Poor performance

**Need further improvement of MCQs stem writing that might have language errors/information:**

For examples: MCQs no………………………………..

**Need further improvement of MCQs distractor writing that might have mis-key/more than one key/implausible.**

For examples: MCQs no………………………………..

**Need further improvement of MCQs distractor writing that might have high number of non-functional distractors (NFD).**

For examples: MCQs no………………………………..

Review all very easy questions so that it can be established that these questions are asking the core knowledge which is essential for all students to have. If not so, that means the distracters are not working well and they will need to be modified in the future exams.

**Supporting Information: S2 Appendix: EXAMINATION FEEDBACK**
